# Impact of probiotic *Veillonella atypica* FB0054 supplementation on anaerobic capacity and lactate

**DOI:** 10.1016/j.isci.2023.108643

**Published:** 2023-12-08

**Authors:** Kristen Gross, Marina Santiago, Joesi M. Krieger, Anthony M. Hagele, Kinga Zielinska, Jonathan Scheiman, Ralf Jäger, Alex Kostic, Chad M. Kerksick

**Affiliations:** 1 Exercise and Performance Nutrition Laboratory, Kinesiology Department, College of Science, Technology, and Health, Lindenwood University, St. Charles, MO, USA; 2 FitBiomics, Inc, New York City, NY, USA; 3 Ludwig Institute for Cancer Research, University of Oxford, Oxford, UK; 4 Increnovo, LLC, Whitefish Bay, WI, USA; 5 Department of Microbiology, Harvard Medical School, Boston, MA, USA; 6 Section on Pathophysiology and Molecular Pharmacology, Joslin Diabetes Center, Boston, MA, USA

**Keywords:** Health sciences, Medicine, Natural sciences, Microbiome

## Abstract

Seven healthy, physically active men (n = 3) and women (n = 4) (30.7 ± 7.5 years, 172.7 ± 8.7 cm, 70.4 ± 11.6 kg, 23.6 ± 4.1 kg/m^2^, 49.2 ± 8.4 mL/kg/min) supplemented for 14 days with a placebo (PLA) or 1 × 10^10^ CFU doses of the probiotic *Veillonella atypica* FB0054 (FitBiomics, New York, NY). Participants had safety panels, hemodynamics, lactate, and anaerobic capacity assessed. Stool samples were collected to evaluate for metagenomic and metabolomic changes. Exhaustion times were not different between groups, whereas anaerobic capacity tended to shorten with PLA (61.14 ± 72.04 s; 95% CI: −5.49, 127.77 s, p = 0.066) with no change with VA (13.29 ± 100.13 s, 95% CI: −79.32, 105.89 s, p = 0.738). No changes in lactate, hemodynamics, or bacterial community changes were observed, whereas 14 metabolites exhibited differential expression patterns with VA supplementation. In conclusion, VA maintained exercise performance that tended to decline in PLA. Supplementation was well tolerated with no changes in safety markers or reported adverse events.

## Highlights

• *Veillonella atypica* FB0054 maintained performance, whereas placebo tended to decrease
• Lactate changes immediately or 5 min after exercise were similar between conditions
• Oral *V. atypica* FB0054 at a dosage of 1 × 10^10^ CFU was well tolerated
• Exercise intensity and duration as key factors to closely evaluate lactate metabolism

## Graphical abstract

**Figure:**
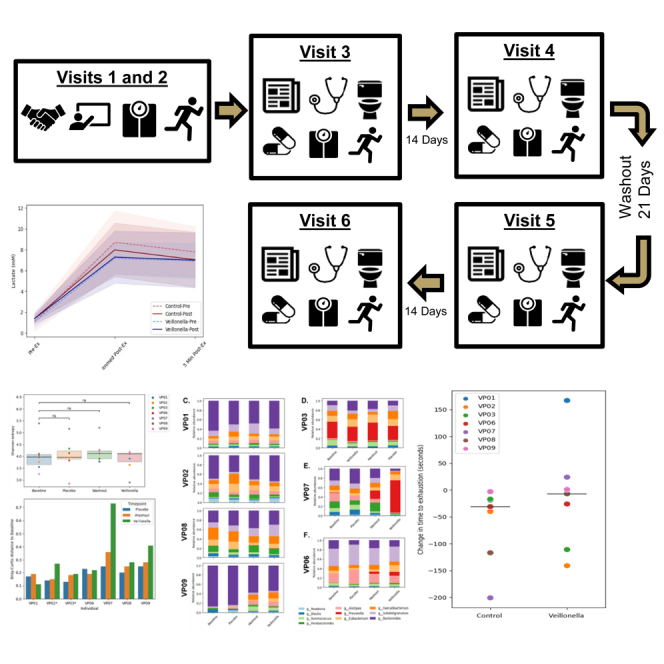

## Introduction

Our health is determined by a complex interplay between our genetics, diet, exercise, lifestyle, and microbiome, and although it is known that diet, exercise habits, and lifestyle can have significant impacts on prevention of chronic disease, the fact that the microbiome can further influence health continues to be explored.^1^ For its part, the human microbiota is a metabolic engine, 100 trillion organisms strong, impacting nearly every physiological system and capable of promoting health or morbidity in numerous diseases.^2^^,^^3^ Physiological responses to exercise are, at least in part, determined by the microbiome.^4^^,^^5^^,^^6^ For example, the absence (via germ-free or gnotobiotic methods) or depletion (via antibiotic cocktail) of the microbiome in animal models diminishes muscle mass^7^^,^^8^^,^^9^^,^^10^ and results in decreased exercise performance.^7^^,^^9^^,^^11^ Furthermore, these phenotypes can be rescued by fecal microbiota transplant from a healthy mouse^8^^,^^9^^,^^10^^,^^12^^,^^13^ or by supplementation with the short-chain fatty acids (SCFAs) propionate or butyrate.^11^^,^^13^^,^^14^^,^^15^ Further, propionate and butyrate, two key metabolites of bacterial fermentation with known health implications, can only be produced by the microbiome and not endogenously by mammalian cells.^16^

Several human studies have identified correlations between microbiome composition and exercise performance, although causation had not been established.^17^^,^^18^^,^^19^ One bacterial genus of interest is Veillonella, which has the ability to convert lactate in the gastrointestinal tract to the SCFA, propionate.^20^ Our recent work demonstrated that the lactate-utilizing genus Veillonella, which is enriched in the gut microbiome of elite marathoners after running a marathon, significantly boosts exercise performance upon colonization in mice in a manner that can be recapitulated by propionate instillation.^14^ The working hypothesis is that as glucose is converted to lactate in the muscle, lactate enters the intestinal lumen via blood circulation, creating a lactate reservoir in the gut. There, lactate can act as a carbon source for microbes like Veillonella. The products of lactate utilization, in this case, propionate, are taken up by the host and utilized as an energy source, which can improve endurance performance. This thesis is significant because it directly couples the metabolic stress of exercise to microbiome activity and then back to the observed exercise response.^14^ Although many commercially available probiotics have been tested for performance improvement with mixed results, *Veillonella atypica* supplementation has not previously been tested for its influence on exercise performance in humans. Here, we conduct a pilot study with the primary aim being to determine the overall clinical safety and influence of *V. atypica* supplementation on running time to exhaustion in healthy adult volunteers. It was hypothesized the *V. atypica* (VA) supplementation would be well tolerated and not be responsible for differences in reported adverse events while also promoting increases in exercise performance.

## Results

### Adverse events

A total of 9 adverse events (nervousness, n = 1; nausea, n = 1; upset stomach, n = 3; stomach cramping, n = 4) were reported throughout the study protocol. Three were reported during PLA (upset stomach, n = 2; stomach cramping, n = 1), and six were reported during VA (nervous, n = 1; nausea, n = 1; upset stomach, n = 1; stomach cramping, n = 3). All reported events were also evaluated for severity where three of the reported adverse events were rated as minor severity and six as a severity of minor-to-moderate. A meta-analysis by Dore et al.^21^ reported increased relative risks of total reported side effect and those adverse events that specifically centered upon gastrointestinal symptoms and abdominal pain in those people who supplemented with a probiotic versus those who supplemented with a placebo. As such, the reported adverse events after VA supplementation of mild-to-moderate severity align with these findings and results of other studies surrounding reported adverse events with probiotic use.

### Randomization

Study attrition resulted in five participants being supplemented with PLA first while two participants supplemented with VA first. To identify if an order effect was present, a categorical variable was created on the assigned order (1 = PLA then VA, 2 = VA then PLA), and all ANOVAs were performed with order as an additional fixed effect to identify if any significant interactions were present. No significant interactions were identified (all p > 0.05) for any of the primary or secondary outcomes.

### Dietary intake

During each visit, participants were questioned by study investigators about any changes in their exercise training and their compliance with the dietary considerations put forth as part of this research study. All participants reported 100% compliance with completing food records and replicating food and drink intake prior to each study visit. Recorded diet records indicated participants consumed 2190 ± 913 kcal per day, 212.5 ± 135.2 g of carbohydrate per day, 113.3 ± 135.2 g of protein per day, and 100.8 ± 49.3 g of fat per day.

### Hemodynamics

Changes in resting heart rate values indicated a significant main effect for time (p = 0.01), no main effect of condition (p = 0.51), and no significant group × time interaction (p = 0.51). The significant main effect was decomposed using paired samples t tests, which revealed that resting heart rate values for PLA did not change (mean difference ± SE: 1.3 ± 9.1 beats per minute; 95% CI: −7.2, 9.7 beats per minute, p = 0.72) in response to supplementation, whereas heart rate values in VA tended to decrease (mean difference ± SE: 5.1 ± 5.8 beats per minute; 95% CI: −0.2, 10.5 beats per minute, p = 0.06, partial eta squared = 0.45). Changes in resting systolic blood pressure indicated no main effect for time (p = 0.88) or condition (p = 0.93), whereas the group × time interaction tended to be significant (p = 0.07). Forced post-hoc comparisons were completed using paired samples t tests to evaluate within-group changes in response to supplementation. Systolic blood pressure values did not change in PLA (mean difference ± SE: −5.0 ± 9.2 mm Hg; 95% CI: −13.5, 3.5 mm Hg, p = 0.20) or VA in response to supplementation (mean difference ± SE: 3.6 ± 15.8 mm Hg; 95% CI: −11.0, 18.2 mm Hg, p = 0.57). The group × time interaction for diastolic blood pressure was not significant (p = 0.15). No significant main effects for time (p = 0.95) or condition were observed (p = 0.84). All hemodynamic data are presented in Table 1.

**Table 1:** Research activities by study visit

	Visit 1	Visit 2	Visit 3	Visit 4	Visit 5	Visit 6
Days	(-7)	(-2)	(0)	(14)	(35)	(49)
Informed Consent Process	X					
Eligibility Review	X					
Peak VO_2_	X					
Body Composition (BIA)		X				
Abstain Caffeine, Alcohol, and Exercise		X	X	X	X	X
Observe Overnight Fast		X	X	X	X	X
Replicate Dietary Intake for 48 Hours		X	X	X	X	X
Anthropometrics (Height [screening only], Weight, BMI)	X	X	X	X	X	X
Heart Rate & Blood Pressure	X		X	X	X	X
Venous Blood (CBC, CMP)			X	X	X	X
Treadmill time to exhaustion at 100% VO_2_Peak	X	X	X	X	X	X
Lactate Responses			X	X	X	X
Stool Collection			X	X	X	X

**Table 2:** Lactate data

Group	Phase of supplementation	Time	Mean ± SD	Time (*p*)
VA	PRE	PreEx (mM)	1.364 ± 0.841	0.001
		IP (mM)	8.821 ± 3.008	
		5 min Post (mM)	7.264 ± 2.487	
	POST	PreEx (mM)	1.636 ± 0.799	<0.001
		IP (mM)	7.379 ± 2.612	
		5 min Post (mM)	6.800 ± 2.668	
PLA	PRE	PreEx (mM)	1.407 ± 0.593	0.003
		IP (mM)	9.050 ± 5.784	
		5 min Post (mM)	6.900 ± 1.732	
	POST	PreEx (mM)	1.707 ± 1.016	<0.001
		IP (mM)	7.900 ± 2.775	
		5 min Post (mM)	6.450 ± 2.727	
Group (*p*)				0.96
Supp (*p*)				0.21
Group × Supp (*p*)				0.91
Group × Time (*p*)				0.78
Supp × Time (*p*)				0.27
Group × Supp × Time (*p*)				0.98

PRE, pre-supplementation; POST, post-supplementation; Group, VA or PLA; Supp, pre-supplementation vs. post-supplementation; PreEx, pre-exercise; IP, immediate post-exercise; 5 min Post, 5 min post-exercise.

### Anaerobic capacity (time to exhaustion)

**Figure 1:**
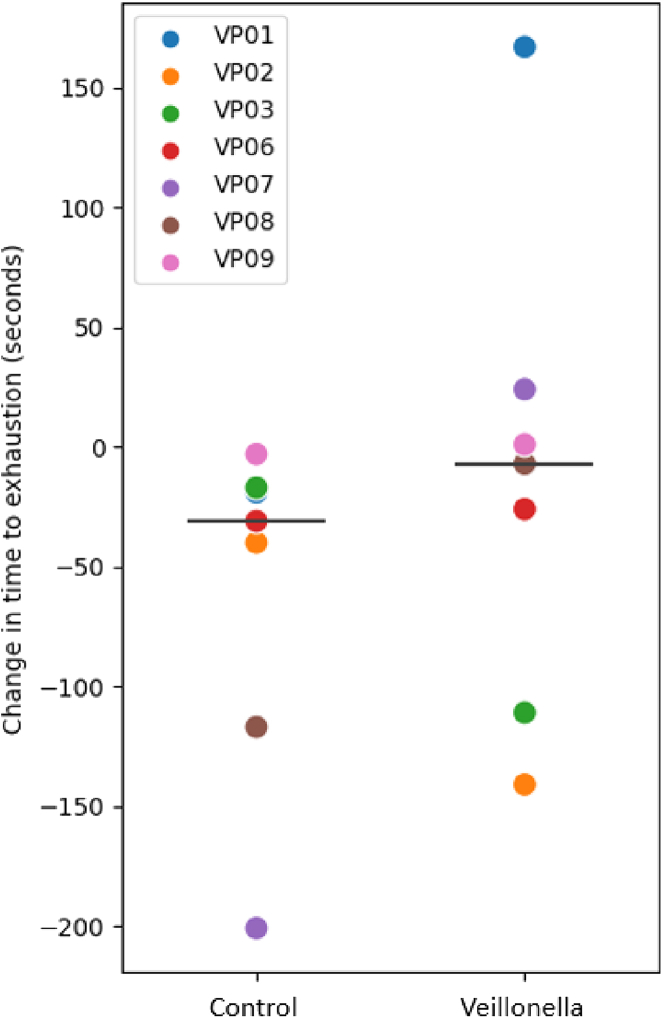
Individual responses for the observed changes in treadmill time to exhaustion for each supplemental condition Control = Placebo (PLA); Veillonella = *Veillonella atypica* (VA). Each respective participant has the same color symbol for each condition. The large horizontal bar is the average change in treadmill time to exhaustion (post- to pre-supplementation) for each respective condition.

No significant group × time interaction was observed for time to exhaustion times (VA-PRE: 346.1 ± 204.6, VA-POST: 285.0 ± 204.3 s vs. PLA-PRE 320.0 ± 317.6, PLA-POST: 306.7 ± 269.6 s, G x T, p = 0.37). Changes in treadmill time to exhaustion performance within each group were assessed in response to supplementation using paired samples t tests. As seen in Figure 1, no changes in treadmill time to exhaustion were found for VA supplementation (mean difference ± SE:13.29 ± 100.13 s, 95% CI: −79.32, 105.89 s, p = 0.738), whereas PLA tended to exercise for a shorter duration, indicating a decrease in exercise performance (mean difference ± SE: 61.14 ± 72.04 s; 95% CI: −5.49, 127.77 s, p = 0.066).

### Lactate changes

**Figure 2:**
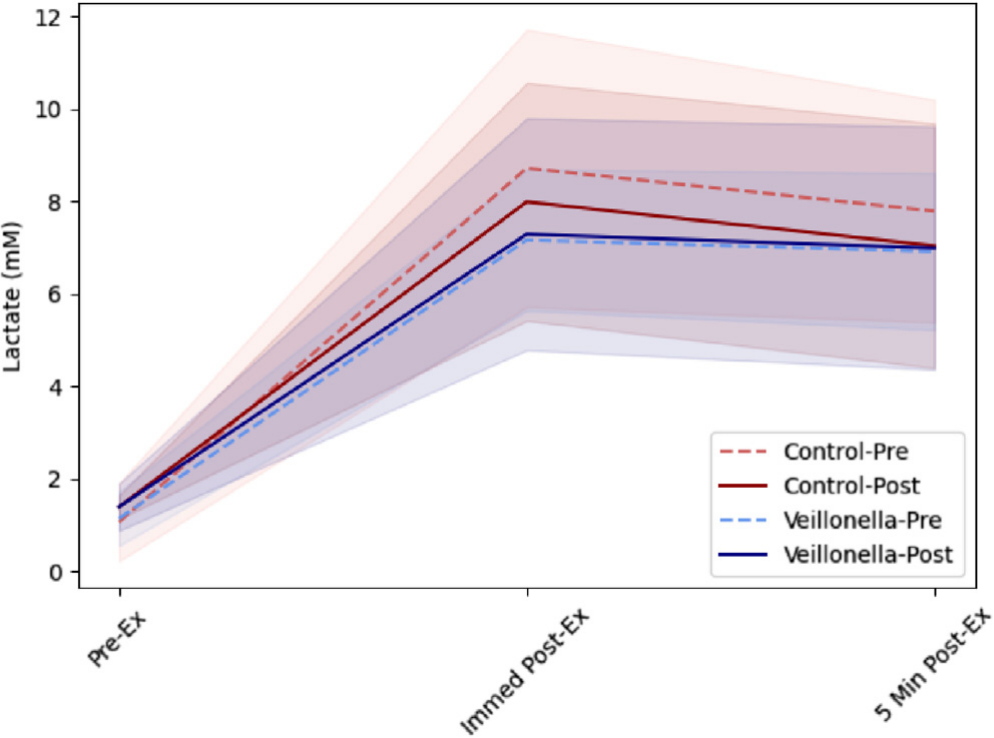
Capillary lactate concentrations in response to time to exhaustion trial at 100%VO2Peak Solid and dotted red lines are associated with the mean changes in control = Placebo (PLA). Solid and dotted blue lines are associated with the mean changes in *Veillonella atypica* (VA). Transparent shading underlaying the mean responses illustrate individual patterns of change for each participant. Pre-Ex = pre-exercise lactate measurement; Immed Post-Ex = immediate post-exercise lactate measurement; 5 Min Post-Ex = 5 minutes post-exercise lactate.

Lactate responses were first evaluated using a group [PLA vs. VA] x supplementation status [Pre-Supplementation vs. Post-Supplementation] x Time [Pre-Exercise vs. Immediate Post vs. 5 min Post] and are provided in Table 2. There was no significant main effect for group (p = 0.96), Supplementation status (Supp, p = 0.21), whereas a significant main effect of time (p < 0.001) was observed. No significant two-way interactions (group x supp, p = 0.91; group x time, p = 0.78; Supp x Time) or three-way interactions (group x Supp x Time, p 0.98) were observed. Lactate responses significantly increased under all Group and Supp combinations (See Figure 2 and Table 2, all p < 0.001).

Separate 2 × 2 mixed factorial ANOVAs with repeated measures on condition were also evaluated to assess between group differences in lactate values collected before, immediately after, and 5 min after completing the time to exhaustion trial. At the pre-exercise time point, lactate values displayed no significant group [PLA vs. VA] x supplementation status [pre-supplementation vs. post-supplementation] interaction (p = 0.96), main effect for supplementation status [pre-supplementation vs. post-supplementation] (p = 0.46), or main effect for condition [PLA vs. VA] (p = 0.75). When each group was looked at individually, no differences were reported between pre-supplementation and post-supplementation pre-exercise lactate values for PLA (p = 0.56) or VA (p = 0.54).

At the immediate post-exercise time point, lactate values displayed no significant group [PLA vs. VA] x supplementation status [pre-supplementation vs. post-supplementation] interaction (p = 0.89), main effect for supplementation status [pre-supplementation vs. post-supplementation] (p = 0.21), or main effect for condition [PLA vs. VA] (p = 0.78). When each group was looked at individually, no differences were reported between pre-supplementation and post-supplementation lactate levels immediately after exercise for PLA (p = 0.11) or VA (p = 0.53).

At the 5 min post-exercise time point, lactate values displayed no significant group [PLA vs. VA] x supplementation status [pre-supplementation vs. post-supplementation] interaction (p = 0.99), main effect for supplementation status [pre-supplementation vs. post-supplementation] (p = 0.38), or main effect for condition [PLA vs. VA] (p = 0.43). When each group was looked at individually, no differences were reported between pre-supplementation and post-supplementation lactate values 5 min after completing the time trial for PLA (p = 0.59) or VA (p = 0.50).

### Hematological and clinical safety markers

All data associated with the hematological and clinical safety markers are provided in Table 3. A significant group × time interaction was observed in the percentage of eosinophils present in the collected samples (p = 0.03). No main effect for time (p = 0.74) or condition (p = 0.26) was observed. When group changes were evaluated separately, the eosinophils percentage significantly decreased in PLA (p = 0.02), whereas no changes were observed in VA (p = 0.19). A significant group × time interaction was observed in mean corpuscle hemoglobin content (p = 0.03). A significant main effect for time (p = 0.04) was observed, and the main effect for condition tended to be significant (p = 0.06). When changes in each group were evaluated separately, the mean corpuscle hemoglobin content in PLA (p = 0.21) did not change, whereas VA values significantly changed (p = 0.01). All values for both eosinophils % and mean corpuscle hemoglobin content stayed within clinically accepted normative values for both variables.

**Table 3:** Hemodynamic, hematological, and clinical chemistry parameters

	Pre	Post	Within (p)	Group × Time (p)
	Mean ± SD	Mean ± SD		
Hemodynamics				
Heart Rate (beats/min)				
PLA	56.8 ± 7.7	55.3 ± 10.0	0.72	0.51
VA	56.3 ± 13.3	51.1 ± 8.8	0.06	
Systolic Blood Pressure (mm Hg)				
PLA	112.4 ± 14.6	117.4 ± 13.1	0.20	0.07
VA	116.4 ± 17.4	112.9 ± 15.0	0.57	
Diastolic Blood Pressure (mm Hg)				
PLA	70.6 ± 7.3	73.1 ± 7.3	0.41	0.15
VA	72.6 ± 7.5	70.3 ± 8.3	0.37	
Hematological Parameters				
Absolute Basophils (cells/μL)				
PLA	43.6 ± 15.9	47.1 ± 26.6	0.66	0.45
VA	44.7 ± 27.6	57.1 ± 23.0	0.06	
Absolute Eosinophils (cells/μL)				
PLA	219 ± 100	195 ± 102	0.12	0.09
VA	145 ± 72	215 ± 124	0.13	
Absolute Lymphocytes (cells/μL)				
PLA	1657 ± 322	1745 ± 366	0.45	0.85
VA	1835 ± 508	1893 ± 437	0.73	
Absolute Monocytes (cells/μL)				
PLA	377 ± 45	401 ± 53	0.24	0.86
VA	348 ± 86	381 ± 67	0.37	
Absolute Neutrophils (cells/μL)				
PLA	2023 ± 565	2227 ± 627	0.50	0.80
VA	2061 ± 348	2183 ± 481	0.41	
Basophils (%)				
PLA	1.02 ± 0.38	1.04 ± 0.64	0.91	0.45
VA	1.02 ± 0.66	1.21 ± 0.48	0.11	
Eosinophils (%)				
PLA	5.12 ± 2.34	4.30 ± 2.49	0.02	0.03
VA	3.38 ± 1.80	4.51 ± 2.47	0.19	
Hemoglobin (g/dL)				
PLA	14.2 ± 0.69	14.1 ± 1.01	0.74	0.57
VA	13.8 ± 0.96	13.9 ± 0.77	0.55	
Hematocrit (%)				
PLA	42.7 ± 2.4	43.2 ± 3.0	0.59	0.39
VA	43.2 ± 2.3	42.5 ± 1.9	0.43	
Lymphocytes (%)				
PLA	38.8 ± 8.8	38.1 ± 7.8	0.82	0.97
VA	40.9 ± 7.6	39.9 ± 7.2	0.51	
Monocytes (%)				
PLA	8.74 ± 1.09	8.81 ± 1.60	0.87	0.76
VA	7.77 ± 0.85	8.06 ± 0.76	0.45	
Mean Corpuscle Hemoglobin (pg)				
PLA	29.9 ± 0.55	29.8 ± 0.82	0.80	0.20
VA	29.6 ± 0.83	29.9 ± 0.64	0.14	
Mean Corpuscle Hemoglobin (g/dL)				
PLA	33.4 ± 0.46	32.7 ± 1.10	0.21	0.03
VA	31.9 ± 0.71	32.8 ± 0.71	0.01	
Mean Corpuscle Volume (fL)				
PLA	89.5 ± 1.40	91.2 ± 3.31	0.17	0.07
VA	92.9 ± 2.92	91.1 ± 2.19	0.01	
Mean Platelet Volume (fL)				
PLA	10.9 ± 0.62	10.9 ± 1.35	0.95	0.83
VA	10.8 ± 1.12	10.9 ± 1.21	0.62	
Neutrophils (%)				
PLA	46.3 ± 10.5	47.8 ± 9.8	0.68	0.60
VA	47.0 ± 8.3	46.3 ± 8.3	0.75	
Platelet Count (Thousand/μL)				
PLA	206.2 ± 18.9	221.6 ± 53.2	0.49	0.87
VA	202.2 ± 47.8	214.7 ± 67.6	0.41	
Red Blood Cell Count (cells x 10^3^/μL)				
PLA	4.76 ± 0.23	4.74 ± 0.36	0.87	0.91
VA	4.66 ± 0.36	4.66 ± 0.22	0.99	
Red Blood Cell Distribution Width (%)				
PLA	12.5 ± 0.39	12.3 ± 0.31	0.03	0.15
VA	12.3 ± 0.43	12.2 ± 0.36	0.51	
White Blood Cell Count (cells x 10^3^/μL)				
PLA	4.32 ± 0.24	4.61 ± 0.59	0.34	0.99
VA	4.43 ± 0.70	4.73 ± 0.73	0.35	
Comprehensive Metabolic Panel				
Albumin: Globulin Ratio				
PLA	1.99 ± 0.38	1.84 ± 0.24	0.18	0.43
VA	1.98 ± 0.35	1.96 ± 0.38	0.80	
Albumin (g/dL)				
PLA	4.37 ± 0.26	4.39 ± 0.22	0.85	0.90
VA	4.40 ± 0.18	4.43 ± 0.25	0.74	
Alanine Aminotransferase (U/L)				
PLA	22.0 ± 11.1	18.7 ± 5.8	0.27	0.78
VA	22.5 ± 16.3	20.4 ± 11.6	0.46	
Alkaline Phosphatase (U/L)				
PLA	48.1 ± 12.6	57.4 ± 19.7	0.13	0.16
VA	51.7 ± 8.0	52.0 ± 11.9	0.93	
Aspartate Aminotransferase (U/L)				
PLA	23.7 ± 8.6	20.1 ± 4.5	0.16	0.68
VA	23.2 ± 11.0	20.9 ± 6.7	0.25	
Bilirubin (mg/dL)				
PLA	0.59 ± 0.20	0.73 ± 0.26	0.17	0.22
VA	0.60 ± 0.08	0.63 ± 0.19	0.65	
BUN: Creatinine Ratio				
PLA	19.0 ± 6.9	18.8 ± 5.6	0.87	0.92
VA	16.9 ± 3.8	16.4 ± 5.0	0.59	
Calcium (mg/dL)				
PLA	9.50 ± 0.28	9.39 ± 0.29	0.44	0.65
VA	9.45 ± 0.26	9.46 ± 0.24	0.96	
Carbon Dioxide (mM)				
PLA	23.9 ± 4.0	26.3 ± 3.3	0.07	0.23
VA	27.2 ± 3.4	26.7 ± 2.0	0.76	
Chloride (mM)				
PLA	106.1 ± 2.3	104.3 ± 1.4	0.13	0.49
VA	106.0 ± 1.8	105.3 ± 1.0	0.36	
Creatinine (mg/dL)				
PLA	0.91 ± 0.12	0.89 ± 0.18	0.55	0.85
VA	0.98 ± 0.24	0.95 ± 0.24	0.53	
Glomerular Filtrate Rate				
PLA	95.6 ± 12.3	99.1 ± 16.3	0.36	0.99
VA	89.8 ± 15.2	93.3 ± 21.0	0.59	
Globulin (g/dL)				
PLA	2.27 ± 0.35	2.40 ± 0.22	0.18	0.52
VA	2.27 ± 0.31	2.31 ± 0.32	0.50	
Glucose (mg/dL)				
PLA	85.0 ± 20.1	78.9 ± 20.6	0.60	0.46
VA	88.2 ± 9.2	88.9 ± 3.1	0.83	
Potassium (mM)				
PLA	4.50 ± 0.29	4.40 ± 0.24	0.35	0.17
VA	4.13 ± 0.31	4.42 ± 0.28	0.18	
Total Protein (g/dL)				
PLA	6.64 ± 0.30	6.79 ± 0.20	0.23	0.68
VA	6.67 ± 0.30	6.74 ± 0.15	0.34	
Sodium (mM)				
PLA	140.4 ± 3.1	140.3 ± 2.0	0.90	0.55
VA	141.5 ± 1.3	140.4 ± 2.1	0.13	
Urea Nitrogen (mg/dL)				
PLA	17.5 ± 7.3	16.9 ± 6.5	0.56	0.95
VA	16.8 ± 6.6	16.0 ± 7.7	0.41	

### Fecal metagenomics and metabolomic analysis

**Figure 3:**
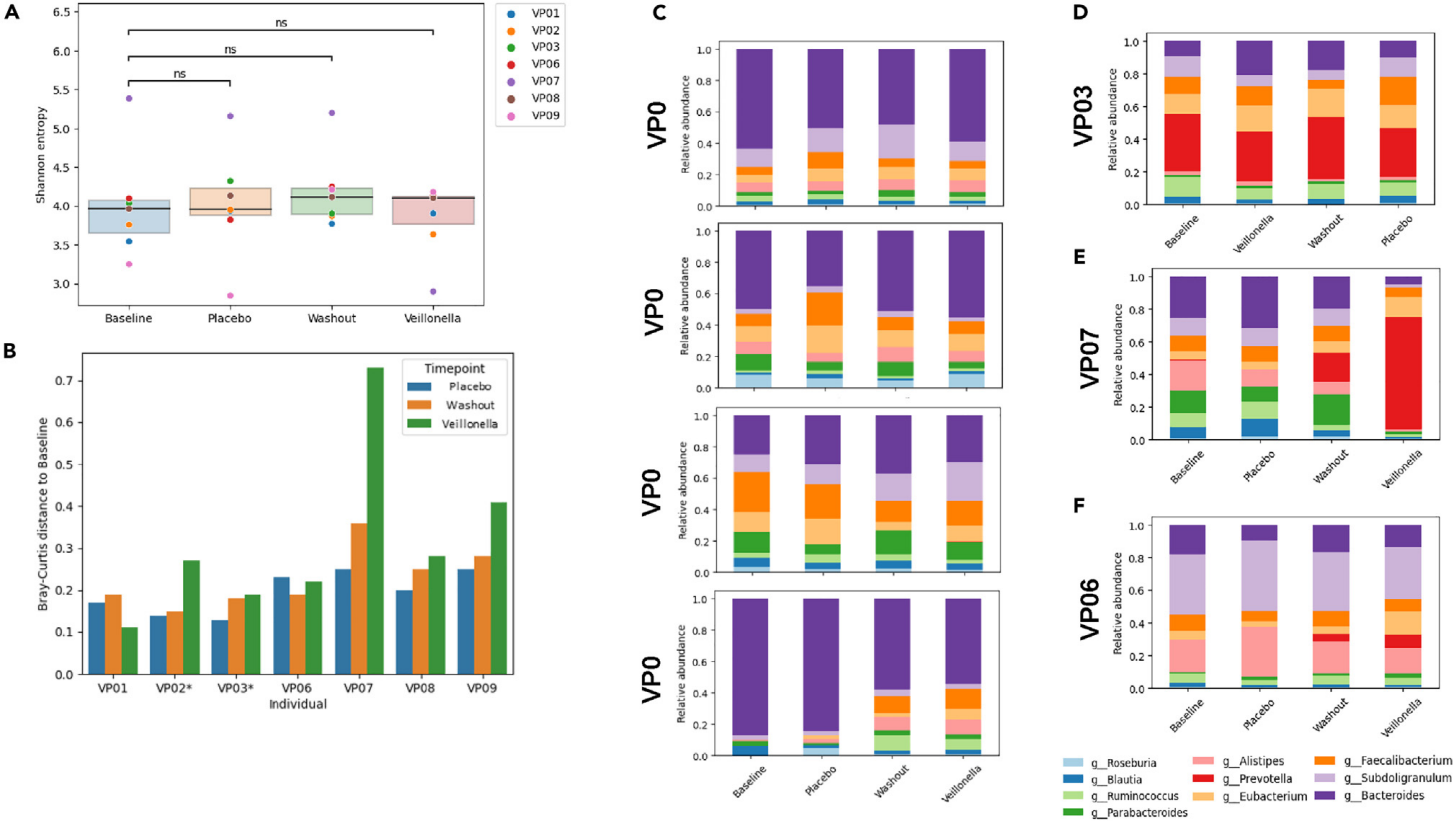
Individual participant microbiome data after baseline, placebo, and *Veillonella atypica* FB0054 supplementation (A) Using independent t tests, no significant changes (all comparisons p > 0.05) in alpha diversity were identified for participants throughout the study. (B) Beta diversity (similarity of samples to other baseline samples) did not change throughout the study, although there were striking increases in beta diversity post-Veillonella in a subset of participants. (C–F) Although there are differences between the microbiome of the participants, there were no significant patterns in terms of changes in the microbiome of each participant. (C) Four participants (VP01, VP02, VP08, and VP09) had Bacteroides as the most abundant genera in their community throughout the study. (D) One participant (VP03) had Prevotella as the most abundant genus throughout the study. (E) One participant (VP07) had large microbiome changes occur during the study. In this participant, the observed bacterial communities at baseline were dominated by Bacteroides and Alistipes, whereas at post-Veillonella treatment, they were dominated by Prevotella. This participant (VP07) also had the greatest improvement in time to exhaustion. (F) One participant (VP06) had Subdoligranulum as the most abundant genera throughout the study.

Metagenomics and metabolomics analyses of all collected stool samples were performed to evaluate how Veillonella supplementation might affect the microbiome community. Shannon entropy was used as a measure of alpha diversity (the richness of the microbiota community in terms of the number and abundance of the different bacterial communities that were present). In general, higher alpha diversity is associated with health, and reduced diversity is associated with a variety of chronic and acute health conditions. There were no significant differences in alpha diversity between the various time points (all p > 0.05), suggesting that the community’s diversity did not change with the supplementation protocol employed (Figure 3A).

**Figure 4:**
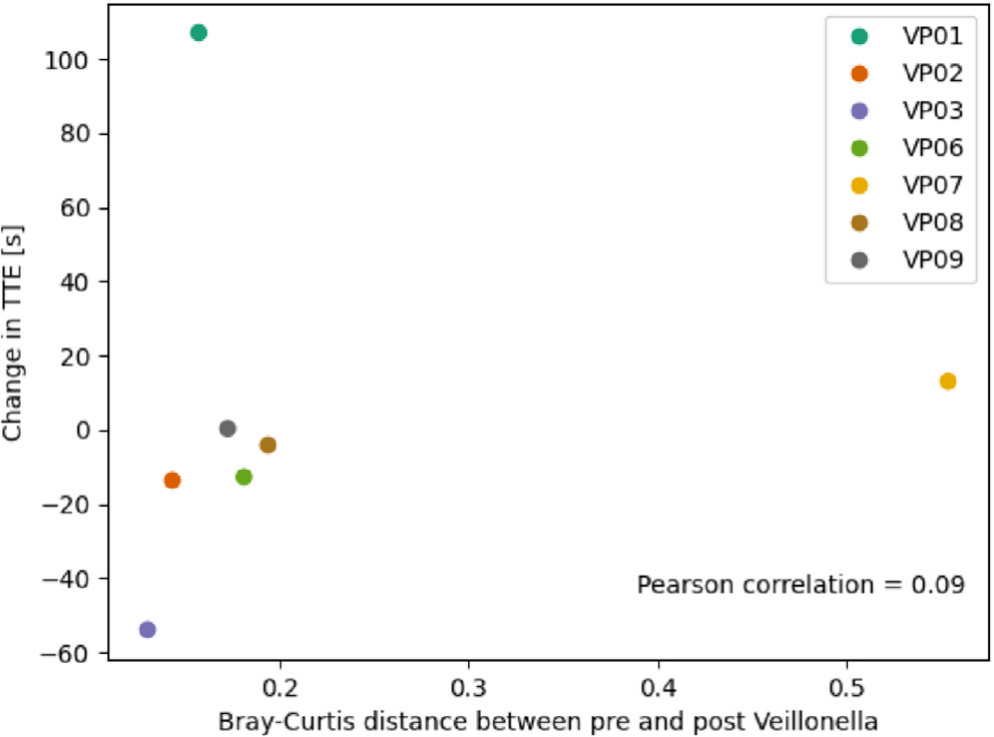
Scatterplot demonstrating beta diversity changes in response to *Veillonella atypica* FB0054 supplementation against changes in time to exhaustion (TTE). Pearson correlations (r) were computed between the change in time to exhaustion and the Bray-Curtis distance between pre- and post-Veillonella.

To further confirm that there are no changes in overall community structure with VA supplementation, beta diversity (similarity of a bacterial community to another bacterial community) was qualitatively evaluated at different time points compared with baseline (Figure 3B). For four participants, limited to no changes in beta diversity were observed over time, although large increases in beta diversity were observed in VP02, VP07, and VP09 after Veillonella supplementation. Overall, no changes in specific taxa or functions throughout the study were observed after placebo use, the washout, or Veillonella use. Pearson correlations were completed to evaluate any potential relationships between changes in beta diversity and changes in time to exhaustion after VA supplementation (Figure 4). Overall, there was no correlation (r = 0.09) for this relationship.

Longitudinal changes in the microbiome community for each participant were assessed to identify potential non-significant patterns, and although the microbiome did change longitudinally in all participants (some with dramatic changes), no discernable associations with VA supplementation were noted.

Metabolomics data provide information on changes in stool metabolites. Variable importance in projection (VIP) scores were used to identify 14 metabolites (Table 4) that were significantly discriminated between Veillonella use and baseline and Veillonella use and placebo but were not changed between placebo and baseline. Many of these metabolites are amino acids, and the majority of the others are metabolites found in foods. Although it is possible that the origin of these molecules is bacterial, and not the diet, the simplest explanation for these results is that these results are due to spurious changes in the participants’ diet (even though participants were told to replicate their diet and indicated 100% compliance in doing so), not due to the action of Veillonella.

**Table 4:** Metabolites that discriminate between Veillonella treatment and baseline and/or placebo

Metabolite	Classification	Δ vs. Baseline (VIP Score)	Δ vs. Placebo (VIP Score)
2-Piperidinone	-Derivative of piperidine (and other natural alkaloids) -Found in foods	None	Decrease (1.96)
Alanine/Sarcosine	Amino acid	Decrease (2.63)	Decrease (1.99)
Aspartic Acid	Amino acid	Decrease (1.24)	None
Betaine	Osmolytes -Found in foods	None	Decrease (3.41)
Cadaverine	Polyamine	Decrease (1.14)	None
Citric Acid	Aerobic metabolism intermediate -Found in foods	Increase (1.68)	None
Glutamine	Amino acid	Decrease (1.42)	Decrease (1.60)
Leucine	Amino acid	None	Decrease (6.13)
Lysine	Amino acid	Decrease (1.32)	None
Nicotinic/Picolinic Acid	Tryptophan metabolite, vitamin B3 -Found in foods	Increase (2.03)	None
Pipecolic Acid	Piperidine derivative (and other natural alkaloids) that assists in absorption of ions in the small intestine -Found in foods	Decrease (2.56)	Decrease (2.15)
Suberic Acid	Derivative of oleic acid -Found in foods	Decrease (1.15)	None
Threonine	Amino acid	Decrease (1.19)	None
Tyrosine	Amino acid	None	Decrease (2.60)

VIP, variable importance in projection scores.

## Discussion

Using a randomized, double-blind, placebo-controlled, crossover manner, we examined the impact of a 14-day supplementation regimen of *V. atypica* FB0054 (VA) on anaerobic capacity (treadmill time to exhaustion), lactate responses to intense exercise, and clinical markers of health in healthy volunteers. The primary findings from this work indicate that a 14-day period of VA supplementation led to no change in anaerobic capacity (treadmill time to exhaustion), whereas PLA supplementation resulted in a tendency for anaerobic capacity to worsen. Supplementation was well tolerated with no clinically meaningful changes in clinical safety biomarkers. This is the first time that a species of live *Veillonella* has been used in a human population. Traditionally, probiotic supplements, the most common of which include lactic acid bacteria and bifidobacteria, have been isolated from food or animal sources.^22^ VA was isolated from elite marathon runners and approved for use in humans through the self-GRAS (generally recognized as safe) pathway, which involved whole genome sequencing and annotation to identify potential threats to human health, stringent toxicological testing,^23^ and approval by an independent committee. This process was affirmed whereby all reported adverse events were mild to moderate in severity (no serious adverse events were reported) and gastrointestinal in nature. Further, the severity and incidence of reported adverse events were consistent with other forms of probiotic supplementation.^21^^,^^24^

**Figure 5:**
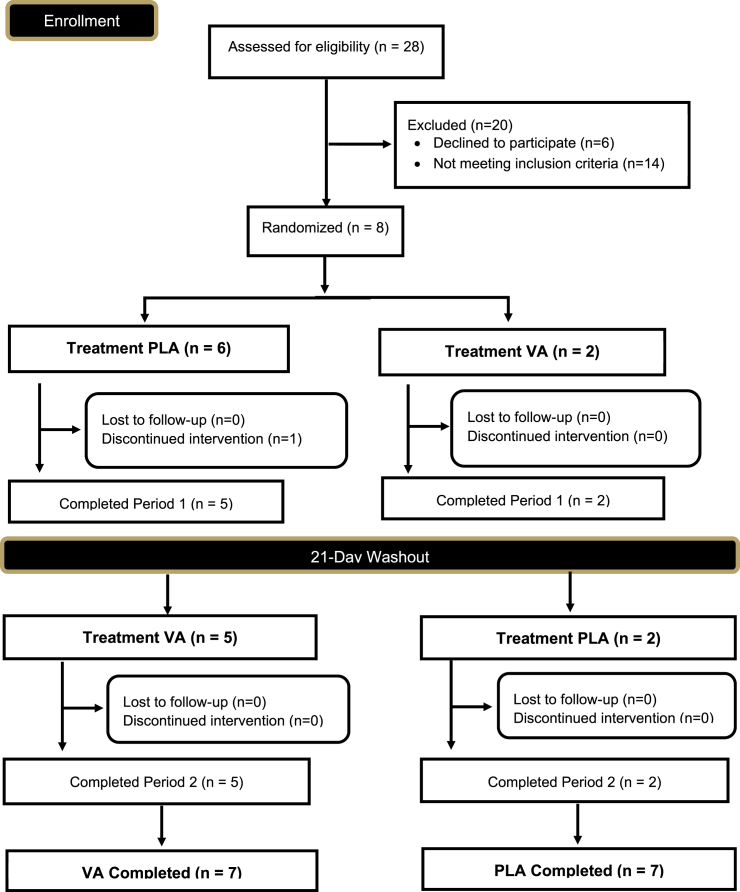
CONSORT diagram

Supplementation with VA did not impact anaerobic capacity, whereas anaerobic capacity tended to worsen with PLA (Figure 5). This finding, although statistically non-significant, does align with previous research involving live probiotics (both single and multi-strain) that have investigated the role of probiotic products on various measures of exercise performance.^25^ For example, Pugh and colleagues^26^ examined the effect of a multi-strain (*Lactobacillus acidophilus* CUL-60, *Lactobacillus acidophilus* CUL-21, *Bifidobacterium bifidum* CUL-20, and *Bifidobacterium animalis* subspecies lactis CUL-34 at a dose 2.5 x 10^10^ CFU/day) probiotic (Proven Probiotics, Port Talbot, UK) on substrate metabolism and exercise performance in seven trained cyclists. No changes in markers of gastrointestinal damage and permeability were observed, but changes in carbohydrate and fat oxidation were observed, whereas time-trial performance was unaffected. *Lactobacillus plantarum* TWK10 is a probiotic strain isolated from Taiwanese pickled cabbage. On multiple occasions,^12^^,^^27^^,^^28^ this strain has demonstrated the ability to improve treadmill run to exhaustion performance and biomarkers associated with fatigue in a dose-dependent manner in a population without a history of regular exercise training. *Lactobacillus casei* Shirota, which is sold as part of yogurt drinks, has been studied in athletes in the context of improving performance through immunomodulation and infection prevention^29^^,^^30^^,^^31^^,^^32^ but has not improved exercise performance. A multi-strain-synbiotic (1 × 10^10^ CFU/day each of *Lactobacillus acidophilus* CUL-21 and *Lactobacillus acidophilus* CUL-60; 9.5 × 10^9^ CFU dose/day of *Bifidobacterium bifidum* CUL-20; and 5 × 10^8^ CFU/day of *Bifidobacterium animalis* subspecies lactis CUL-34, *Bifidobacterium bifidum* CUL-20, *Bifidobacterium bifidum* CUL-20, and *Bifidobacterium lactis* CUL-34; BioAcidophilus Forte, Biocare Ltd., Birmingham, UK) has been tested for the ability to decrease endotoxin levels and increase performance in recreational athletes.^33^ While endotoxin levels did decrease significantly, mean run times between groups exhibited a strong tendency to improve, but ultimately failed to reach statistical significance, in the probiotic group versus placebo. Finally, Strasser et al.^34^ reported in trained athletes that supplementation with a multi-strain probiotic (1 x 10^10^ total CFU per day of *Bifidobacterium bifidum* W23, *Bifidobacterium lactis* W51, *Enterococcus faecium* W54, *Lactobaccilus acidophilus* W22, *Lactobacillus brevis* W63, and *Lactococcus lactis* W58; Ecologic Performance, Winclove B.V., Amsterdam, The Netherlands) reduced the incidence of upper respiratory tract infections but did not benefit athletic performance.

In the context of these mixed results and the pilot nature of our study alongside its small sample size, the results of this study are not unexpected. Many challenges exist when examining the ability of probiotics to influence exercise performance.^24^ As a starting point, choosing the right endpoint is a significant challenge. Based on previous *in vivo* work and the anticipated mechanism of action of VA, we chose a run-to-exhaustion model to assess performance. However, in the placebo group, we saw a decrease in time to exhaustion over time, when we would expect to see no change. This unanticipated observation may be due more to the underlying exercise psychology whereby untoward or desirable feelings associated with completion of intense exercise are rewarded by terminating participation. In addition, the study design required four separate experimental visits to the laboratory where the time to exhaustion protocol was included (five including the familiarization trial), thus the mental fatigue surrounding completion of the exercise test may have impacted motivation on subsequent tests. Importantly, we randomized the order of treatment administration to avoid an order effect, which was confirmed in a separate three-way ANOVA performed on the time to exhaustion data. We purposefully chose an exercise intensity (100% VO_2_Peak) that would generate significant production of lactate, but in hindsight, this intensity may have been too high for a few key areas. First, it is well established that cardiac output is redistributed away from the viscera to provide more blood to the working muscle. This physiological response would have led to a significant reduction in blood flow and ultimately limited the ability of VA to metabolize the lactate and offset performance. Thus, we feel that the high exercise intensity may have pushed our participants too quickly and aggressively into anaerobic metabolism, which simply may not have allowed enough time for VA to metabolize lactate. For these reasons, we feel a longer bout of exercise at a challenging (but not maximal) intensity would increase circulating lactate across a longer period and thus would create a richer environment for VA to metabolize to various metabolites. While more work is needed to further verify this consideration, previous research using the TWK10 strain (which has consistently demonstrated ergogenic outcomes) has utilized lower running intensities (60%–70% VO_2_Peak) for longer periods of time.^12^^,^^27^ Along these lines, we have previously posited that it may not be the lactate production and subsequent consumption by metabolic tissues that effects endurance so much as the production of propionate, a short-chain fatty acid energy source for the human host, which could increase endurance. Previous *in vitro* and *in vivo* models have linked propionate to mitochondrial changes^35^^,^^36^ and based upon these findings, selecting a longer exercise bout may have allowed more time for propionate to be produced and exert its potential metabolic or ergogenic potential. Certainly, more research is needed to fully explore this possibility.

Beyond the exercise task that was selected, choosing the right population with the appropriate baseline microbiome composition may change how the probiotic affects the host. The composition of the microbiome is determined by host genetics, immune system, diet, lifestyle, and the microbes already resident in the gastrointestinal tract.^2^^,^^3^ The ability for any probiotic strain to exert a positive effect on the host is dependent on all these factors. In this work, we studied a population that was already aerobically fit, lean, and physically active. Thus, it is possible our sample may have already had Veillonella present in the gut, reducing the need for VA supplementation. To this point, analysis of the bacterial communities observed in our collected fecal samples (Figure 3) did not appear to change in response to VA or PLA supplementation. Notably, this may also have been an explanation for the lack of changes observed for exercise performance. In a more sedentary population or a population with low relative abundance of *Veillonella*, we might expect to see more improvement in endurance and a greater dynamic range of response compared with placebo.

An additional key consideration was our selection and timing of biomarker assessment. We assessed capillary lactate before, immediately after, and 5 min after completing the run to exhaustion. Using this approach, we observed no change in capillary lactate levels between the placebo and VA conditions. In considering this further, one must realize that the majority of lactate is recycled through the liver and resulting plasma concentrations depend upon participant training status and style as well as the intensity and duration of completed exercise and potential differences in the presence and activity of monocarboxylate transporters, which move lactate in and around various tissues.^37^ These factors alone highlight the difficulty of tracking changes in lactate in response to open-ended exercise tasks as the duration upon which the lactate is being produced has the potential to vary quite a bit between participants and even within participants due to open-ended exercise tasks exhibiting much higher coefficients of variations and lower reliability.^38^ This was anticipated and explains why participants practiced the time to exhaustion two times prior to their first actual time to exhaustion trial. The key point nonetheless is made that more variation within and between exercise performance will ultimately impact the lactate kinetics during the exercise bouts. Although lactate levels commonly peak approximately 5 min after intense exercise, further consideration of our results against the proposed mechanism highlights the potential need for future investigations to consider lactate measurements for longer periods of time after exercise completion. To this point, evaluation of lactate 15, 30, and 60 min after completion of the exercise bout may have afforded better opportunity to evaluate if VA supplementation was able to metabolize the available lactate.

As mentioned previously, no changes were observed in the bacterial communities assessed in our collected stool samples after performing metagenomic and metabolomic evaluations (Figure 3). Directly related to VA supplementation, widespread changes in the microbiome were not anticipated as *Veillonella* is a small intestinal bacteria found in relative low abundance in our stool. After supplementation, we would anticipate that VA would engraft in the small intestine (not the large intestine). As a result, large changes in the biome would be surprising after the addition of one species of bacteria. A small number of stool metabolites significantly discriminated between post-Veillonella use and baseline or post-placebo. The majority of these were amino acids (Table 4), and the others were associated with foods. Thus, any associations between these metabolite changes and VA supplementation may be spurious diet-related changes and not specifically microbiome-mediated metabolite changes.

In conclusion, this pilot study of probiotic *V. atypica* FB0054 in humans shows supplementation did not improve endurance performance but did seem to be able to offset performance decrements observed in the PLA condition without any difference in lactate responses between supplementation. In addition, supplementation of the strain within the confines of our prescribed supplementation regimen (1 × 10^10^ CFU daily dose for 14 days) appears to be safe and tolerable. Due to the small pilot nature of our project and the small observed effect on our assessed performance effect, readers are encouraged to view our conclusions with a preliminary mindset. Additional research is needed to explore FB0054, the ergogenic potential of *V. atypica*, more fully as changes in the population assessed, intensity of exercise employed, and using a closed vs. open-endpoint exercise test could also yield different outcomes.

### Limitations of the study

The results of this study are limited by the small sample size. That being said, our chosen sample size aligned with other intervention studies involving probiotics using crossover^26^ and parallel^27^ designs, but the need for a larger small sample size would have helped to further strengthen the observed outcomes. For the current study, each participant supplemented for 14 days with a three-week washout. The duration of the supplementation regimen was selected based on results observed from two recent investigations by our research group involving a similar study design and probiotic supplementation^39^^,^^40^ as well as a recent literature review on probiotics Mohr and colleagues.^41^ However, it should be highlighted that the majority of the investigations reported in this review administered probiotics strain(s) for longer periods of time than we did in the present study, thus a longer supplementation period using this strain may have impacted our outcomes. A final factor impacting our outcomes may have been the diverse range of ages in our participants as aging has been shown to impact the composition and quantity of various microbes found in our guts.^42^ Although a full understanding of how age may impact how the gut responds to exercise remains to be fully established, it seems reasonable to highlight that recruiting a cohort of individuals with a narrower age range may have helped reduce any confounding impact age had on our observed outcomes. Finally, challenging exercise is well established to impact gut permeability,^43^ and many studies have demonstrated probiotics ability to improve gut permeability.^25^ While participants were asked to self-report adverse events and the common side effects connected to poor gut permeability, future research should more closely explore changes in biomarkers associated with gut permeability.

## STAR★Methods

### Key resources table

**Table:** 

REAGENT or RESOURCE	SOURCE	IDENTIFIER
**Biological samples**		

RNA*later™ Stabilization Solution*	Fischer Scientific	Cat# AM7020
OMNIgeneGUT	DNAgenotek	Cat# OMR-200
Vacutainer® EDTA Tubes	BD	Cat# 366643
Vacutainer® SST™ Tubes	BD	Cat# 367988
Lactate Plus Meter Test Strips	Nova Biomedical	Cat# 40813

**Critical commercial assays**		

Complete Blood Count (including Differential and Platelets)	Quest Diagnostics	Test Code 6399
Comprehensive Metabolic Panel	Quest Diagnostics	Test Code 10231
DNeasy PowerSoil Pro Kit	Qiagen	Cat# 47014
Quant-iT™ PicoGreen™ dsDNA Assay Kits and dsDNA Reagents	Thermo Fisher	Cat# P7589

**Software and algorithms**		

OWUS-4.3 Windows O2 Uptake Software	Parvo Medics	https://www.parvo.com/trueone-2400/
Automated Self-Administered Dietary Assessment Tool (ASA24)	NIH	https://asa24.nci.nih.gov/
MetaPhlAn2	PMID: 26418763	https://github.com/biobakery/MetaPhlAn2
HUMAn2	PMID: 30377376	https://github.com/biobakery/humann
MetaKey	ProDigests	https://prodigest.eu/metakey/
Xcalibur Software v4.0.27.21	Thermo Fisher	https://www.thermofisher.com/us/en/home/industrial/mass-spectrometry/liquid-chromatography-mass-spectrometry-lc-ms/lc-ms-software/lc-ms-data-acquisition-software/xcalibur-data-acquisition-interpretation-software.html
SIMCA® v17	Sartorius	https://sartorius.com/en/products/process-analytical-technology/data-analytics-software/mvda-software/simca
QIIME2	https://qiime2.org	RRID:SCR_021258
SPSS v27	IBM	https://www.ibm.com/spss?lnk=flatitem
GraphPad Prism	Dotamatics	https://www.graphpad.com/features

**Other**		

Informed consent was obtained from all subjects involved in the study. The study was conducted in accordance with the Declaration of Helsinki, and approved by the Institutional Review Board of Lindenwood University.	Lindenwood University & Declaration of Helsinki	Protocol #22–60, approval date: 1/4/22.
Woodway Desmo-Evo Treadmill	Woodway	https://www.woodway.com/home-page/
Polar H10	Polar USA	https://www.polar.com/us-en/sensors/h10-heart-rate-sensor
TrueOne 2400 Metabolic Cart	Parvo Medics	https://www.parvo.com/trueone-2400/
InBody 570	InBody	https://inbodyusa.com/products/inbody570/
Lactate Plus Meter	Nova Biomedical	62624
NovaSeq 6000	Illumina	6000
Vanquish Quaternary Pumping System	Thermo Fisher	VF-P20-A
Q-Exactive™ Quadrupole-Orbitrap High-Resolution Mass Spectrometer	Thermo Fisher	IQLAAEGAAPFALGMBDK
Fecal metagenomic sequence data	Sequence Read Archive (SRA) https://www.ncbi.nlm.nih.gov/sra/	PRJNA103161

### Resource availability

#### Lead contact

Further information and requests for resources and reagents should be directed to and will be fulfilled by the lead contact, Chad Kerksick (ckerksick@lindenwood.edu).

### Materials availability

No materials were used in this study.

#### Data and code availability

(1) Human metagenomic sequence data have been deposited at Sequence Read Archive (PRJNA1031161) and are publicly available as of the date of publication. Accession numbers are listed in the key resources table. Fecal metabolomics data is posted on Zenodo at DOI: https://doi.org/10.5281/zenodo.10139375. Deidentified human data reported in this paper will be shared by the lead contact upon request.
(2) All original code has been deposited at Zenodo (DOI: https://doi.org/10.5281/zenodo.10139375) and is publicly available as of the date of publication. DOIs are listed in the key resources table.
(3) Any additional information required to reanalyze the data reported in this paper is available from the lead contact upon request.

### Experimental model

This pilot study was conducted using a randomized, double-blind, crossover study design. A pilot approach was considered to establish proof of concept in humans for oral *Veillonella atypica* administration to be deemed healthy and well-tolerated and to examine *Veillonella atypica*’s ability to augment exercise and lactate responses. Healthy men and women (n = 7) between the ages of 18–50 years of age participated in this study. As seen in Table 1, after being screened for eligibility and providing consent (Lindenwood University Institutional Review Board, protocol # 22–60, approval date: 1/4/22), participants had their VO_2_Peak determined. After observing a brief (15–20 min) break, eligible participants practiced their first time to exhaustion trial at 100% VO_2_Peak. Study visit 2 was scheduled approximately 3–7 days after study visit 1, and participants had their body composition assessed for descriptive purposes before completing a second time to exhaustion running trial to provide further familiarization to the exercise bout. Prior to each of the next four study visits (visits 3–6), participants collected a stool sample and upon arrival at the laboratory had their body mass measured before having a venous blood sample collected for assessment of complete blood count and comprehensive metabolic panel. After blood collection, participants completed a standardized treadmill walking and jogging warm-up followed by a 3-min resting period before assessing anaerobic capacity by completing a treadmill time to exhaustion test with a speed set where 100% VO_2_Peak occurred during the previous visit. Capillary (fingertip) lactate levels were assessed before, immediately after, and 5 min after completion of the exhaustion trial. After completing visit 3, all participants were randomized in a double-blind, placebo-controlled, crossover fashion, to orally ingest for 14 days either a placebo (corn starch) or a 1 × 10^10^CFU dose of *Veillonella atypica* FB0054 (VA) (Fitbiomics, Inc. New York). After completing 14 days of supplementation, participants returned to the laboratory to complete an identical battery of tests as what was completed during study visit 3. After completing study visit 4, participants observed a 21-day wash-out period before returning to the laboratory to complete study visit 5. After completing study visit 5, participants were crossed over to complete a 14-day supplementation regimen with the remaining study supplement before returning for study visit 6. Figure provides a graphical overview of our study design and Table 1 provides a detailed layout of what assessments were completed at each study visit. All study visits were generally scheduled between 0600 and 1000 h with each visit being scheduled at a near identical time. Prior to each visit, an overnight fast (food, caffeine, and nicotine) was observed and participants refrained from any vigorous exercise for at least 24 h prior to each visit. Prior to study visit 3, participants completed a two-day food and fluid record. This log was copied, and study participants were instructed to replicate their food and fluid intake prior to study visits 4–6. All blood samples were centrifuged for 15 min at 3000 rpm and all plasma was removed and stored at −80°C. Stool samples were collected by each study participant following instructions provided by the research team, frozen at their residence and subsequently stored at −80°C. This study protocol was retrospectively registered on ClinicalTrials.org as NCT05816291 on April 13, 2023.

**Figure:**
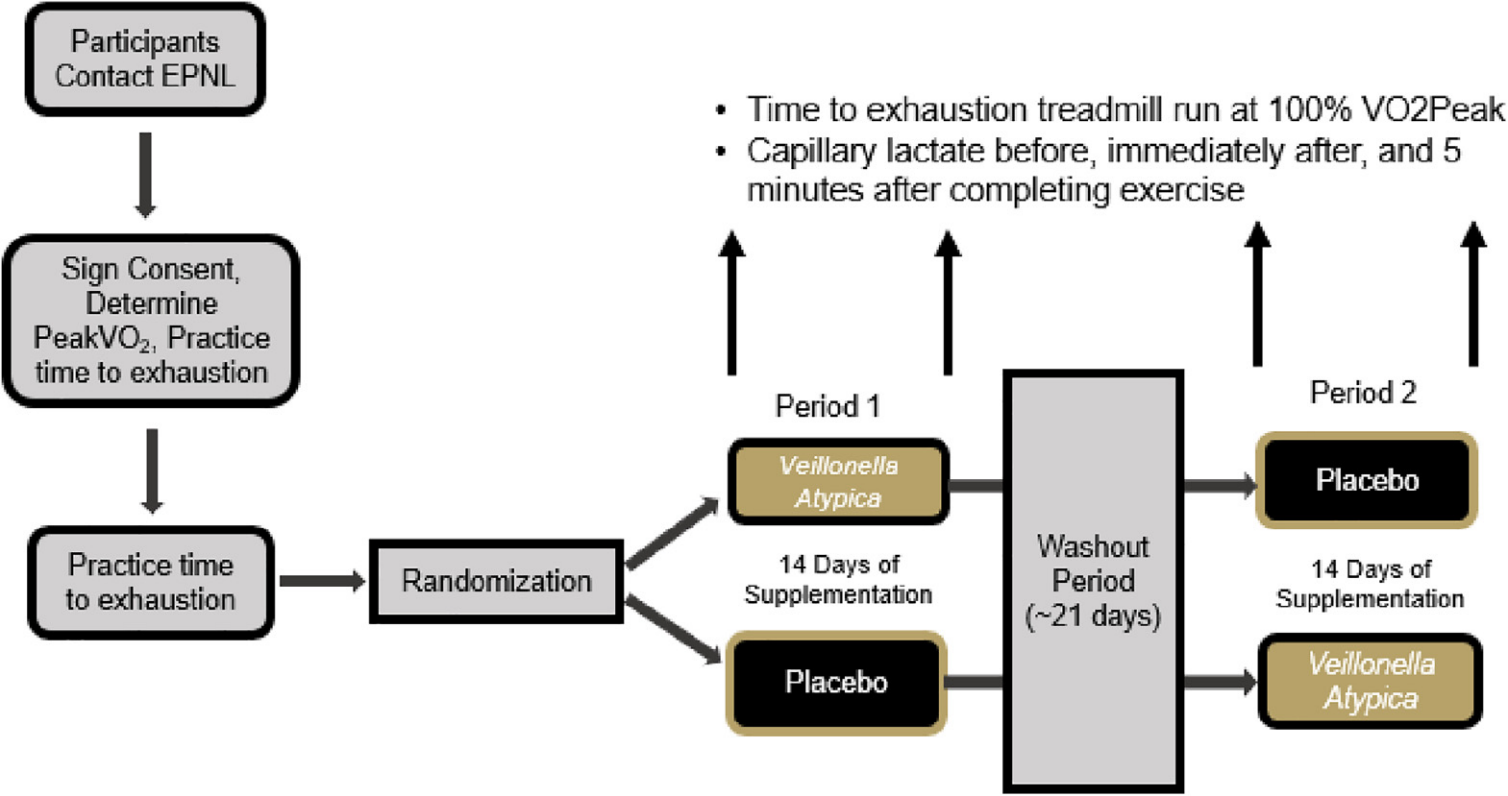
Overview of research design

#### Study participant details

Seven healthy Caucasian men (n = 3) and women (n = 4) (30.7 ± 7.5 years, 172.7 ± 8.7 cm, 70.4 ± 11.6 kg, 23.6 ± 4.1 kg/m^2^, 19.9 ± 9.2% fat, 49.2 ± 8.4 mL/kg/min) completed all aspects of the study protocol. Sample size for this study was estimated using the previous findings of Carter and Jeukendrup,^44^ which employed a near identical study design. Using these parameters, a sample size of seven participants alongside a small (*d* = 0.3) to moderate (*d* = 0.4) effect size and an alpha level of 0.05 was estimated to yield a statistical power of 0.65–0.70. Study participants recruited for this study reported getting 6.7 ± 0.8 days of physical activity per week and 5.4 ± 1.5 days of exercise per week. To be included, participants needed to: provide their consent, be between 18 and 50 years of age, complete regular exercise at least 150 min per week, be normotensive (systolic <140 mm Hg, diastolic <90 mm Hg), and have a body mass index between 18.5 and 29.9 kg/m^2^ or a body fat % < 30% fat. Participants were excluded if they were currently being treated for any medical condition, consumed more than two alcoholic drinks per day or ten drinks per week, currently smoking or quit within the past six months, currently following a ketogenic diet, pregnant, or lactating, attempting to lose weight, or do not participate in some form of aerobic exercise at least two days per week. All participants who provided initial consent were randomized into the trial and completed all aspects of the research investigation. A CONSORT diagram has been included as Figure 5.

### Method details

#### Procedures

##### Anthropometrics and hemodynamics

Participants had their standing height and weight determined using a standardized wall-mounted stadiometer (Tanita, model # HR-200, Tokyo, Japan) and a self-calibrating digital scale (Tanita, model # BWB-627A Class III, Tokyo, Japan). Body mass index (kg/m^2^) was then calculated. Resting heart rate and blood pressure was assessed using an automatic blood pressure monitor (OMRON, model # Hem 907XL, Kyoto, Japan) after participants sat quietly for three to 5 min.

##### Body composition

Body composition was assessed for descriptive purposes using a bioelectrical impedance analyzer (InBody 570, Beverly Hills, California). Participants were required to observe an overnight fast between 0600 and 1000 h by trained research personnel according to device specifications.

##### Dietary records and hydration control

Participants completed a three-day food and fluid log prior to study visit 2. Food logs were submitted using the National Institutes of Health Automated Self-Administered 24-h dietary recall (ASA24).^45^ Each participant was provided with login information and detailed instructions on how to accurately submit the food logs through the ASA24 software. A copy of this food record was then provided to each participant, and they were instructed to replicate their diet prior to each study visit for the remainder of the study. Strict hydration controls were not employed due to the nature of the study, intensity of exercise effort, and time of blood sampling. As such, *ad libitum* water intake was allowed and made consistent across visits. Notably, no participants consumed water prior to commencing the warm-up to the treadmill exhaustion protocol and between the final lactate measurement.

##### Fecal collection

Within 24 h prior to beginning and ending each supplementation period, participants collected a fresh stool sample. Participants were provided with all materials necessary to collect, store, and transport the stool specimen including commode fecal collection system, fecal collection tubes with screw top lids and scoops attached, disposable gloves, freezer packs, specimen labels, and transfer containers. Participants were instructed, if possible, not to urinate into the collection container. Participants then extracted a representative stool sample and filled each of the two provided sample collection tubes. All samples were immediately submerged in preservation reagents (RNA Later and OMNIgene) and frozen at −20°C. Tubes were then secured, labeled, and frozen. All remaining stool was discarded into the toilet and flushed. Participants were instructed to keep their samples in the freezer and to remove them at the latest possible time to minimize thawing during transfer to the laboratory for storage. Upon arrival in the laboratory each sample was immediately stored at −80°C.

##### Peak oxygen consumption

Exercise testing was completed on a Woodway Desmo-Evo treadmill (Woodway USA, Inc., Waukesha, WI USA). During familiarization, participants completed a graded, staged treadmill protocol to assess peak oxygen consumption following the protocol of Camic et al.^46^ The protocol began with a 1-min warm-up walking at 3.0 mph at 1.0% grade. The protocol then utilized 2-min stages starting at 5.0 mph and 1.0% grade. Upon completion of each 2-min stage, treadmill speed progressively increased 1.0 mph until 9.0 mph was reached. At that point, treadmill speed was maintained at 9.0 mph and grade was first increased to 2.5% with progressive increases in grade of an additional 2.5% upon completion of each 2-min until volitional fatigue was reached. Heart rate was monitored continuously throughout the test (Polar H10, Polar, Kempele, Finland). VO_2_Peak was achieved if the respiratory exchange ratio (RER) reached >1.05 and heart rates were within ten beats of the age-predicted maximal heart rate (Max HR = 208 – [0.7 x age]). VO_2_ was averaged over 15-s intervals and VO_2_Peak was reported as the highest VO_2_ value recorded throughout the exercise test. As previously reported,^46^ the test-retest reliability for testing using this protocol indicated the intraclass correlation coefficient was R = 0.95 with a standard error of measurement being 97 mL/min, with no significant difference being observed between the mean differences of the test and re-test values.

##### Anaerobic capacity (running time to exhaustion)

On five separate occasions, participants had their anaerobic capacity evaluated by completing a treadmill time to exhaustion protocol. Briefly, participants walked or jogged at a self-selected speed for 3 min as a warm-up. After observing a 3-min rest period, treadmill speed was set at the speed upon which 100% VO_2_Peak was achieved. Participants were instructed to run at this speed for as long as they could. No feedback in terms of duration, pacing, etc. was provided, and participants were provided verbal encouragement to run for as long as possible. The same research team members were present during each test and all team members were instructed to provide similar feedback and encouragement between visits for each participant. All testing visits were scheduled at similar times to avoid differences in motivation, fatigue, and diurnal characteristics.

##### Venous blood collection, processing, and analysis

At the beginning and ending of each supplementation condition, study participants had venous blood collected from a forearm vein using standard phlebotomy techniques into two ethylenediaminetetraacetic acid coated (EDTA) Vacutainer tubes and two serum separating tubes (SST). The collected blood samples were gently inverted ten times before allowing serum tubes to clot at room temperature. These samples were then placed into a chilled container and sent to a commercial diagnostic laboratory (Quest Diagnostics).

##### Capillary lactate collection and analysis

In response to completing the time trial at 100% VO_2_Peak during each supplemental condition, capillary lactate was collected from a fingertip. Prior to collection, each finger was quickly sterilized with an alcohol wipe and wiped clean and dry. A sterile lancet was used to pierce the skin and a small drop of blood (∼10 μL) was collected and placed onto an analysis strip. All capillary samples were collected using a handheld lactate analyzer (Lactate Plus, Nova Biomedical Corp., Waltham, MA). Capillary blood samples were collected using these methods before, immediately after, and 5 min after completing the time to exhaustion trial. Two samples were collected at each time point and the calculated reliability between measurements during this protocol was CV = 11%.

##### Complete blood count

Whole blood samples were collected in EDTA tubes and analyzed at a commercial diagnostic laboratory (Quest Diagnostics) for changes in red blood cell count, white blood cell count, platelet count, hemoglobin, hematocrit, red blood cell dimension width (RDW), mean corpuscle volume (MCV), mean corpuscle hemoglobin (MCH), mean corpuscle hemoglobin content (MCHC), neutrophil % and cell count, lymphocytes % and cell count, monocytes % and cell count, eosinophils % and cell count, and basophils % and cell count (granulocytes → neutrophils, eosinophils, basophils).

##### Comprehensive metabolic panel

Serum samples were collected into serum separating tubes (SST) and assayed at a commercial diagnostic laboratory (Quest Diagnostics) for a comprehensive metabolic panel (albumin, albumin/globulin ratio (calculated), alkaline phosphatase, alanine aminotransferase (ALT), aspartate aminotransferase (AST), blood urea nitrogen (BUN), BUN/creatinine ratio (calculated), calcium, carbon dioxide, chloride, creatinine with estimated GFR, globulin, glucose, potassium, sodium, total bilirubin, and total protein).

##### Supplementation protocol

Subjects were assigned to ingest one of two supplement conditions in a randomized, double-blind, and crossover fashion. A randomized order of treatments was generated by random.org by a blinded research team member. The same number of capsules were provided to each participant. Each capsule was identical in color, shape, smell, and transparency. Participants were instructed to consume their assigned with 8–12 fluid ounces of cool water. Participants supplemented for 14 consecutive days with either a placebo (corn starch) or a 10 billion (1 × 10^10^ CFU) dose of VA. This dose was chosen as a result of internal consumer research and aligns with dosages delivered (10^7^–10^11^ CFU/g according to previously published reviews^21^^,^^41^ involving probiotic supplementation and other probiotic supplementation studies which employed a similar crossover design.^39^^,^^40^ Participants returned their supplement container at subsequent study visits and compliance was calculated. Compliance to the supplementation protocol was calculated at 100%.

##### Fecal metagenomics

DNA was extracted using the DNeasy Powersoil kit and standard protocols. Shotgun metagenomics analysis of stool samples was performed at Diversigen. Samples underwent Diversigen’s standard quality control (PicoGreen ds DNA quantification) and library preparation. Samples were sequenced on an Illumina NovaSeq 6000 using a 2x150 bp flow cell with a mean target depth of 20 million reads.

##### Metagenomics data processing and analysis

DNA sequences were taxonomically classified using the MetaPhlAn2 analysis tool.^47^ To create filtered taxonomy tables, samples with fewer than 10,000 sequences were discarded, and the number of counts for each operational taxonomic (OTU) was normalized to the OTU’s genome length. Functional profiling was carried out using the HUMAnN2 analysis pipeline.^48^ The functional tables were filtered to the same subset of samples as the filtered taxa tables. Univariate statistics were used as well. Values below the limit of quantitation (LOQ) were replaced by half of the lowest detected relative abundance above the LOQ. We used Qiime2^49^ to calculate microbiome diversity measures and participant microbiota community profiles. Code for metagenomics analysis is available upon request. Data is made publicly available on SRA (accession # PRJNA103161).

##### Fecal metabolomics

Metabolomics was performed on ProDigest’s MetaKey global polar metabolomics platform. Raw stool samples were subjected to a solid-liquid based extraction protocol [19,20] and subsequently lyophilized. Lyophilized material was weighed (50.0 ± 1.0 mg) and dissolved in ultrapure water that contained internal standards. After shaking the resulting mixture and performing subsequent centrifugation, the supernatant was collected and filtered through a polyvinylidene fluoride filter (0.22 μm pore size). From the purified extract, a 10-μL aliquot was injected into the ultra-high performance liquid chromatography high-resolution mass spectrometry (UHPLC-HRMS) system.^18^^,^^19^ Chromatographic separation was achieved on a Vanquish quaternary pumping system (Thermo Fisher Scientific, USA), equipped with an Acquity HSS T3 C18 column (1.8 μm, 150 × 2.1 mm) (Waters Corporation, UK). A binary solvent system consisting of ultrapure water (solvent A) and acetonitrile (solvent B), both acidified with 0.1% formic acid, was used at a constant flow rate and by applying a gradient profile. Detection was performed on a Q-Exactive standalone bench top quadrupole-Orbitrap high-resolution mass spectrometer (Thermo Fisher Scientific, USA), which was preceded by heated electrospray ionization (HESI-II source) in polarity switching mode. The instrument was operated at a resolution of 140,000 full width at half maximum and in full-scan mode (m/z scan range of 53.4–800 Da), meaning that all detected ions were registered whereby no fragmentation was applied.

##### Metabolomics analysis

Metabolites were identified and quantified using Xcalibur software version 4.0.27.21 (Thermo Fisher Scientific, USA) was used. In general, a metabolite was considered below the LOD/LOQ if the metabolite’s observed peak area was below 1,000,000 arbitrary units. Following peak integration, the area ratio was determined for each metabolite by calculating the ratio between the area of the metabolite and that of the most suited internal standard. For the applied multivariate statistical analysis, unsupervised principal component analysis (PCA X) and supervised orthogonal partial least squares discriminant analysis (OPLS-DA) modeling were performed using SIMCA version 17. Metabolites of interest included those that significantly distinguished between post-VA and post-placebo and/or post-VA and baseline metabolite profiles but did not distinguish between baseline and post-placebo profiles. Metabolomics data and code is available upon request.

##### Adverse event reporting

The occurrence of adverse events was recorded throughout the entire duration of the study using spontaneous reporting by the study participants, clinical evaluation, or interaction of a research team member with a study participant or through of a study participant’s research file. All recorded events were systematically categorized using MedDRA system organ class and lowest level terms (LLT) before being graded using Common Terminology Criteria for Adverse Events (CTCAE), v5.0, US Dept Health & Human Services (published: November 27, 2017).

### Quantification and statistical analysis

All statistical analysis was completed in a blinded fashion. Primary endpoints for this study were treadmill time to exhaustion and lactate responses to the time to exhaustion trial. Secondary endpoints included hemodynamic responses and tertiary outcomes were changes in hematological, clinical safety, and adverse event outcomes. All data is presented as means ± standard deviations and analyzed using IBM SPSS 27 (Armonk, NY USA) and graphs were generated using GraphPad (La Jolla, CA) or custom python scripts (Python 3.9.13, numpy 1.21.5, pandas 1.4.4., seaborn 0.11.2). A p value of 0.05 was used to make all statistical determination and any p value between p = 0.051–0.10 was considered a statistical trend. Differences in Shannon diversity were determined by the Kruskal-Wallis test. Normality was assessed using Shapiro-wilk normality tests. Within-within (condition x time) factorial ANOVAs with repeated measures on condition and time with Least Significant Difference adjustments for multiple comparisons were used to assess differences between conditions at each individual time point for time to exhaustion, hemodynamics, all clinical safety parameters, and lactate changes. If the assumption of heteroscedasticity for repeated measures were violated, a Greenhouse-Geisser correction factor was applied. Paired samples t-tests were used to compare differences before and after supplementation for each condition. Grubbs outlier tests were completed on all data to evaluate the presence of any statistical outliers that may otherwise impact the reported outcomes. Pearson correlations were completed to evaluate the strength of any relationships.

### Additional resources

This study protocol was retrospectively registered on ClinicalTrials.org as NCT05816291 on April 13, 2023.
